# Behaviour change intervention for smokeless tobacco (ST) cessation delivered through dentists within a dental setting: a feasibility study protocol

**DOI:** 10.1038/s41405-022-00104-w

**Published:** 2022-04-21

**Authors:** Shaista Rasool, Richard Holliday, Zohaib Khan, Fiona Dobbie, Linda Bauld

**Affiliations:** 1 Usher Institute, University of Edinburgh, Edinburgh, Scotland; 2 Institute of Public Health and Social Sciences, Khyber Medical University, Peshawar, Pakistan; 3 School of Dental Sciences, Faculty of Medical Sciences, Newcastle University, Newcastle upon Tyne, England

**Keywords:** Dental public health, Tobacco cessation in dentistry

## Abstract

**Objectives/Aim:**

To adapt a structured behavioural support intervention for smokeless tobacco (ST) cessation and to assess the feasibility and acceptability of delivering the intervention via dentists within dental settings in Pakistan.

**Material and methods:**

The study will have 3 phases: (1) Adapt a previously developed intervention to make it suitable for delivery in a clinical/dental setting through qualitative interviews with dental patients and dentists; (2) A multi-centre, pilot randomised control trial in two teaching dental hospitals in Pakistan. Participants (dental patients) will be randomly assigned to intervention or control group in a 1:1 allocation ratio to receive either a structured behavioural support intervention involving face to face counselling or self-help material plus usual care. Each participant will be in the study for approximately 6 months and the overall study is expected to run for 12 months; (3) An embedded qualitative process evaluation. All trial participants will be followed up at 3 and 6 months to assess self-reported ST use. Outcome measures will include: rates of eligibility, recruitment and retention, randomisation group cross-over rates, the provision of data on ST use behaviour, fidelity to the intervention and qualitative assessment of acceptability and feasibility.

**Discussion:**

Despite the high use of ST in Pakistan, users are offered negligible cessation support. The findings of this multi-centre, mixed-method feasibility study will inform the scope for a larger trial on ST cessation delivered through the existing dental health system.

## Introduction

More than 85% of the global smokeless tobacco (ST) users live in the South and South-East Asian region [1, 2]. The use of ST in Pakistan, a South-Asian country, has deep-rooted cultural and traditional ties. ‘Pan’, largely considered as an occasional delicacy, is often a part of the festive celebrations and is consumed by young and old, irrespective of gender. ‘Naswar’, the most popular ST product in Pakistan, is sold openly to minors in transparent bags, without any health warning [3]. Despite the alarmingly high rates of use and evidence on the associated health harms, such as oral cancers [4], oral pre-malignant lesions [5] and fatal strokes [4], ST control in Pakistan remains largely neglected.

While many tobacco users wish to quit, the majority fail to do so. Dependence on tobacco makes it challenging to quit and support for cessation is often minimal in most countries [6, 7]. According to the ‘Global adult tobacco survey’ GATS Pakistan (2014), 21.1% of the ST users made a quit attempt in the past year. In more than half of the cases, this was an un-assisted quit attempt (50.8%). Of the 43.3% of ST users and 42.9% of tobacco smokers who visited a health care provider, only 33.7% ST users and 51.8% of the tobacco smokers were offered quit advice by healthcare providers respectively [8]. These figures are clearly reflective of the gap between the need and availability of cessation support offered to tobacco users (especially ST users) in Pakistan.

Despite substantial evidence on the effectiveness of affordable tobacco cessation interventions at both the individual and population level [9, 10], progress in implementing Article 14 of the Framework Convention on Tobacco Control (FCTC), pertaining to tobacco cessation, has been slow, especially in low and middle income countries (LMICs) [11]. Despite being a signatory of the FCTC since 2005 [12], Pakistan lags behind its neighbouring countries in terms of ST control laws and alignment of the existing laws with FCTC guidelines. ST control remains largely neglected against the backdrop of the challenges that Pakistan faces with implementation and enforcement of key measures for smoked tobacco [3, 13]. ST products in Pakistan are not taxed, the contents are not regulated and the users are offered negligible cessation support.

Like many LMICs, Pakistan lacks resources to effectively address the multiple health problems it faces, which is one of the main challenges in treating tobacco dependence [13, 14]. However, the existing health system (such as dental settings) can be effectively engaged to extend its role to tobacco control with minimum investment [15]. This multi-centre mixed-method feasibility study aims to adapt a behaviour support intervention for ST cessation and assess the feasibility and acceptability of delivering the intervention via dentists within dental settings in Peshawar, Pakistan.

## Material and methods

### Study design

We plan to conduct a mixed-method feasibility study with an incorporated multicentre, randomised, controlled pilot study. The Randomised Control Trial (RCT) protocol conforms to the Standard Protocol Items: Recommendations for Interventional Trials (SPIRIT) and the Consolidated Standards of Reporting Trials (CONSORT) statement for reporting RCTs. The aim of this study is to adapt a previously developed structured behaviour support intervention for ST use cessation and assess the feasibility and acceptability of delivering it in dental hospitals via dentists.

The study will be conducted in three phases. In phase 1, qualitative work will be undertaken to inform the adaptations required to the intervention resources, to prepare the training workshop and module for the participating dentists and to inform the trial design. The intervention that will be adapted and tested in this study, is a structured, theory-based behavioural support intervention for ST cessation, known as BISCA, ‘Behavioural support Intervention for Smokeless Tobacco Cessation in South Asians’. BISCA is a behavioural support intervention for ST cessation which was originally developed in the UK for South-Asian community [16]. The intervention was piloted in four NHS Stop Smoking Services (Leicester, Leeds, Bradford and Tower Hamlets) in the UK (amongst ST users of South Asian origin) and in a community setting in Karachi, Pakistan and was found feasible and acceptable to deliver [16]. It has also been adapted to meet the needs and cultural context of ST users in Bangladesh, India and Pakistan [17].

While the intervention has been tested in community settings with ST users in South-Asia, it has not been tested in hospital/clinical settings in this region. Before delivering it in routine dental practice via dentists it is important to understand the views of dental patients who are users, on their ST use initiation, continued ST use, cessation attempts and their views on the intervention resources. It is also important to understand the range of issues influencing delivery and integration of behavioural support in routine dental practice and to make necessary adaptations to make it suitable for delivery in routine dental practice. The aim is to retain the core components relating to the effectiveness of the intervention, whilst making appropriate changes that will enhance its acceptance in a dental/clinical setting. For this purpose, interviews with dentists working at the study sites and dental patients ‘who use ST’ and are visiting the study site will be undertaken.

In phase 2, the intervention will be delivered through a multi-centre, randomised, controlled pilot trial in which participants (dental patients who are ST users) will be randomly allocated to two arms: intervention and control (self-help material) with 1:1 allocation ratio. Phase 3, will involve an embedded qualitative study, within the pilot trial, after the intervention delivery is complete, to gain in-sight into the views of the trial participants regarding the feasibility and acceptability of the intervention and the trial procedures. The project is expected to run for approximately 12 months.

### Trial registration

ISRCTN: Study ID ISRCTN18072109; 10.1186/ISRCTN18072109 registered 13. January, 2022. Data collection started on 23, October 2021.

### Study sites

This study will be carried out in Peshawar, which is the capital city of Khyber Pakhtunkhwa (KP), the north-western province of Pakistan, where the use of Naswar is most common. The study will be conducted at two specialised tertiary level, dental teaching hospitals in Peshawar: a public sector facility, the Khyber College of Dentistry (KCD); and a private sector facility, the Sardar Begum Dental College (SBDC). Being the only public sector specialized dental hospital in Peshawar, KCD caters to most of the urban and rural population of Peshawar as well as nearby areas.ST use has been linked to low socioeconomic status and since services are offered at nominal rate at KCD, it is believed that most of the patients visiting this hospital belong to low socioeconomic class. It is hoped, that the choice of this site will make patient recruitment easier. In order to explore how intervention delivery varies across public and private hospitals, SBDC has been selected as the 2nd study site. Both the study sites have expressed their support for the study in writing by issuing ‘letter of support’ signed by the deans of the hospitals.

### Participants and sampling

*Phase 1:* Qualitative research does not aim to seek findings that are representative. Therefore, in line with good practice recommendations for qualitative research, a non-probability sampling approach will be adopted for purposively selecting dentists and patients. Dental patients and dentists will be purposively selected from the selected hospitals. We anticipate interviewing 10–12 dental patients and 10–12 dentists. The reason behind this sample size for dentist’s interview is, that we aim to interview the senior- most dentist from each of the five specialities of dentistry, from each of the two study site (prosthodontics, periodontics, oral medicine, operative dentistry and maxillofacial surgery). The choice of interviewing senior faculty is based on the assumption that, they would have immense experience of treating dental patients and hence would have had a fair experience of interacting with dental patients who are ST users; for whom the intervention is to be delivered. Furthermore, senior faculty are the administrative heads of their speciality, which is why these dentists are responsible for deciding the standard operating procedures for their respective departments. It is also believed that their views would be reflective of the entire department that they run. The criteria for dental patients will be, those patients visiting the study site(s) for a dental treatment and are current ST users. The reason behind the anticipated sample size for the patient’s interviews is that we aim to recruit 5–6 patients from each study site. These will be purposively selected to ensure there is the sample diversity that qualitative research requires. This means that we recruit across both genders and different age groups ranging from (18–78 or more years) using an age bracket of 15 years and therefore taking at least one patient from each 15 years bracket, from each study site. The reason for including different age groups is based on the assumption that, the users belonging to different age groups will have varying degree of exposure to ST because of the length of habit. In most cases, users start using ST in early adulthood and over the years their habits change. Regarding gender, while we aim to interview female respondents, based upon evidence from previous research, we are not very optimistic on being able to do so. No female ST users were interviewed in a recent qualitative study on ST cessation in Peshawar [17]. While the use of ST is common, it is not common for women in Peshawar to disclose this. Therefore, we anticipate a female representation of 20% (from any age bracket).

*Phase 2 and 3:* The trial participants will be recruited from the periodontology and prosthodontics departments of the selected hospitals. The choice of recruitment of dental patients from periodontology and prosthodontics is based on practical as well as biological grounds. ST use, is a potential risk factor for periodontal changes, such as gingival recession [18], attachment loss [19, 20], which can contribute towards tooth loss. Therefore, it is believed that a substantial number of patients using ST, will be visiting these departments. Furthermore, in most cases, patients referred to these departments require multiple visits for the completion of their treatment. However, if we are unable to recruit the required sample size within the stipulated time, then recruitment will be done from the department of ‘operative dentistry/endodontics’ as well.

Participants will be dental patients visiting SBDC/KCD, for a dental treatment and who are 18 years and above, are regular ST users, are willing to visit the study site for three visits and willing and able to provide written or thumbprint informed consent. Patients who are currently accessing cessation support or/and are unable/unwilling to provide written or thumbprint informed consent will be excluded. The intervention will be delivered by consenting, licensed dentists, working as faculty members or post-graduate trainees, at the selected wards/department of KCD and SBDC. These dentists will be trained in intervention delivery prior to the recruitment of patients.

In line with good practice recommendations, no formal sample size calculation has been completed for this pilot study [21]. Recommendations suggest, sample sizes of 30 or more participants in each arm [22, 23]. We anticipate an attrition rate of 36% from a similar feasibility trial [16] and hence, aim to recruit 50 participants in each arm.

An embedded qualitative process evaluation (phase 3) will be undertaken for which some of the trial participants and participating dentists will be invited, for face-to-face interviews, after completion of all trial visits. The purpose of these interviews is to qualitatively assess, the acceptability and feasibility of the intervention. A non-probability sampling approach will be used and at this stage it is anticipated that 10–12 patients who participated in the trial will be interviewed. Taking 5, 6 patients from each study site and at least 1 patient from each of the 15 year age bracket, from 18–78 years; as stated above in phase 1. As for the dentists, we anticipate interviewing 10–12 dentists who participated in the trial. It is anticipated that at least, 2–3 dentists will participate from each department, since we propose to conduct the trial in two departments at each study site, providing approximately 8–12 dentists in total. We aim to interview all dentists who participated in the intervention delivery. Equal number of respondents will be selected from each of the two study sites.

### Recruitment and consent

*Phase 1:* All new patients at KCD and SBDC are first examined at the outpatient department (OPD). A brief history (including history of tobacco use) is taken by the dentist on duty, to reach a preliminary diagnosis, before referring the patient to the relevant department/ward for treatment. The first author will be present in the OPD and will brief the dentists working in the OPD about the study. Potential participants (patients) will be identified by the dentists, during history taking while working in the ‘OPD’ in the presence of the first author. Participants identified at the OPD will be invited by the first author to participate in the study. Senior dentist, from the five selected departments, will be invited face-to face by the first author to participate in this phase of the study. The study protocol will be discussed in detail and written informed consent will be sought, at least 24 h prior to the interviews.

**Fig. 1:**
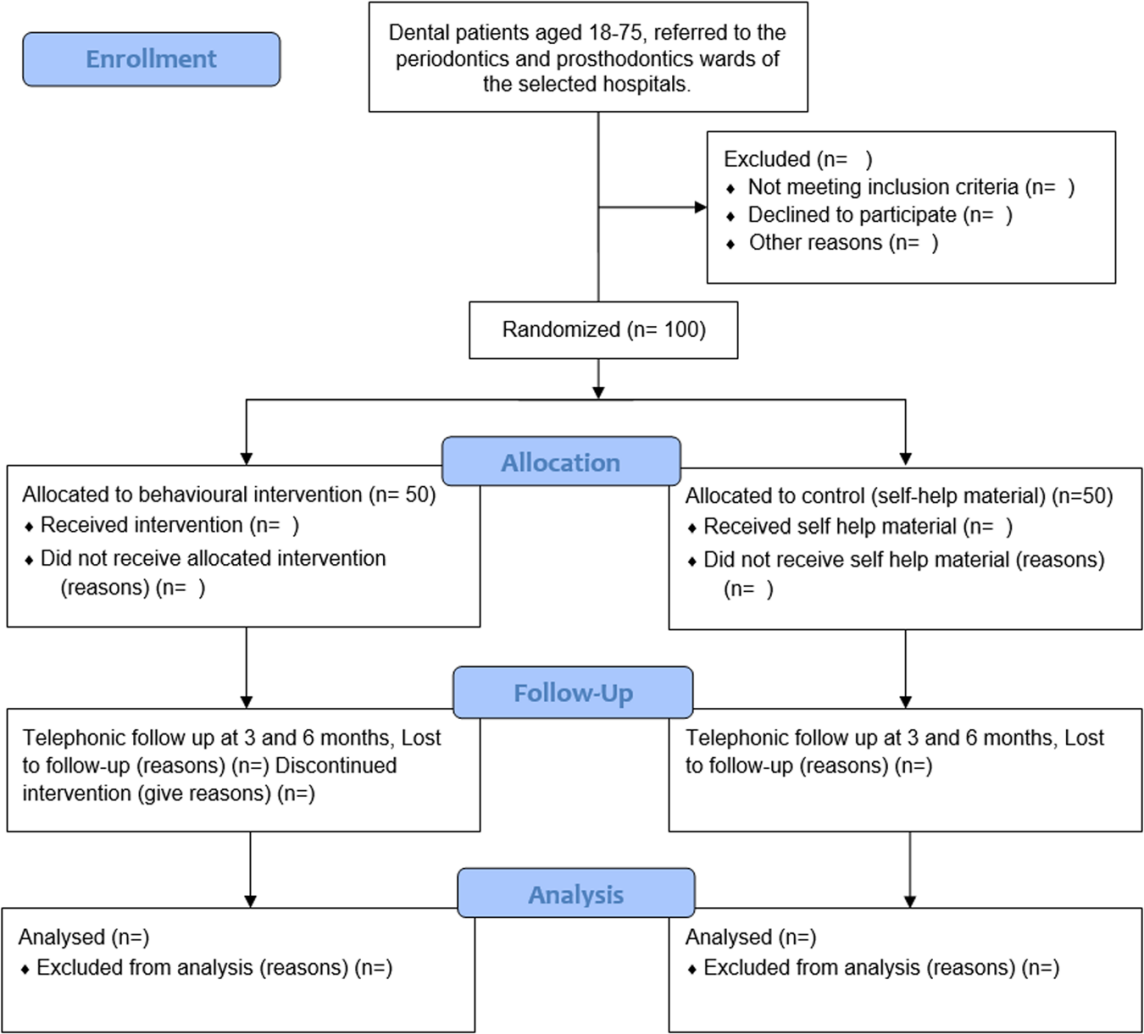
Participants flow through the study.

*Phase 2 & 3:* The overall trial flow is outlined in Fig. 1. Potential participants for the pilot RCT, will be identified from the selected departments of the two dental hospitals. A pro-forma is filled for every new patient in the department, ‘which includes a detailed history of habits’ to reach a final diagnosis, before deciding the treatment plan for the patient. Potential participants will thus be identified by the participating dentists. Once the participants are identified, a member of the research team will discuss the study and invite them into the trial, before seeking written or thumbprint informed consent. All permanent faculty members and trainee medical officers, working in the selected wards will be invited, face-to-face by the first author, to participate in the trial for delivering the intervention. A copy of the signed informed consent form will be given to all trial participants(patients and dentists) for their records.

### Intervention group

Participants in the intervention group will receive an adapted version of the structured behaviour support intervention for ST cessation, ‘BISCA’. While the intervention will be adapted upon completion of phase 1, a TIDieR checklist for the intervention prior to adaptation is provided (Table 1). BSICA has been developed by identifying key theory-based determinants, that govern early initiation and continued use of ST, in users of South-Asian origin. These determinants have been mapped to behaviour change techniques (BCTs) that are effective in behaviour change in general and in tobacco cessation in particular [24]. These BCTs have then been translated into the ‘intervention activities’ and further developed into a culturally appropriate resource to facilitate the delivery of these activities. It comprises 23 activities across three sessions. These include, providing information for raising awareness of ST harms and the benefits of quitting, boosting motivation and self-efficacy, and developing strategies to manage triggers, withdrawal symptoms, and relapse [16, 17]. BISCA will be delivered by dentists in three sessions namely; pre-quit, quit and post-quit sessions. All sessions will involve face to face counselling with the aid of a flipbook which contains interactive messages for the participants to view on one side facing the patient, whereas, the other side has prompts for the dentists to guide the conversation with the patients. The pre-quit session will be delivered on the participant’s first visit for their dental treatment. The aim of this session is to assess and develop the participants’ intention to change and to plan and prepare the participant for quitting. In this session, a ‘quit date’ will be set with the participant, which will be the date that the participant, agrees to quit tobacco. This date will coincide with the participants’ next dental visit. Participants will be given a take-home booklet (a copy of interactive cards) in this session, the purpose of which is to reinforce the messages delivered in the session. The ‘quit session’ will be delivered to the participants on their subsequent dental visit (2nd visit for their dental treatment). The aim of the quit session is to support the participant on the quit day. Focus in this session will be on withdrawal symptoms and managing triggers. Participants will be given a calendar for self-monitoring purpose in their 2^nd^ session, which they have to mark for the number of days that they have remained abstinent/or not. They will be asked to bring this calendar in their next visit. The third visit, ‘the post quit session’ will be scheduled alongside the third visit of the participant for treatment. The purpose of this session is to provide ongoing support and confirmation of abstinence. While we cannot be sure how long each session will last, based upon past experience of delivering BISCA in a community setting, the first sessions are expected to last for 4–54 min, the 2^nd^ session is expected to last 5–35 min and the last session is expected to last for 2–22 min.

**Table 1:** TIDieR Checklist for the intervention-BISCA.

S. No.	Item	Definition
1	Brief name	Behavioural support Intervention for Smokeless Tobacco Cessation in South Asians (BISCA).
2	Why	Focused face-to-face behavioural support for tobacco cessation is very effective in increasing quit rates. Dentists are the first to notice any changes in the oral mucosa due to tobacco use. Most of the treatments require more than one visit. These factors place dentists in a unique opportunist position to identity tobacco users and offer quit advice and support for tobacco cessation. It is recommended that provision of quit advice and support by dentists should be integrated into their routine clinical practice.
3	What (materials)	*Participants (users):* Self-help calendars and take home booklet for the patients. The purpose of the take-home booklet is to reinforce the messages delivered in the sessions. Whereas, the calendar will be given for self – monitoring purpose ‘offered at the quit-session’. *Intervention providers (dentists*): Practice manual and interactive cards will be given to the participating dentists.
4	What (procedures)	Structured behaviour support ‘face-to-face counselling’ will be delivered by dentists in three sessions namely pre-quit, quit and post-quit sessions. *Pre-quit session*. The activities included in the pre-quit session are aimed to develop the users’ intention to change and to plan and prepare the user for quitting. *Quit session*. The activities included in this session are centred on a reflection on the preparation and support on the quit day. *Post quit session:* The purpose of this session is provision of ongoing support and confirmation of abstinence.
5	Who will Provide	Dentists trained in delivering BISCA.
6	How	BISCA will be delivered as an individual face to face conversation.
7	Where	Periodontology and Prosthodontics departments of KCD and SBDC.
8	When and How much.	BISCA will be delivered in three sessions. Each session will be delivered before or after the dental treatment is completed (as deemed appropriate by the dentist).Based upon past experience of BISCA in a community setting, the first sessions are expected to last for 4–54 min, the 2^nd^ session is expected to last 5–35 min and the last session is expected to last for 2–22 min. The sessions will scheduled one or two weeks apart.
9	Tailoring	The conversation will be tailored to the specific type of ST product used by the patient. Furthermore, the quit advice in the quit-session and continued support offered in the subsequent session will be guided by the level of patients’ motivation and engagement. The duration of the sessions will be dependent upon the interaction between the dentists and patient.
10	Modification	N/A
11	How well (planned)	Two ‘half-day’ workshop will be arranged to train all participating dentists to deliver BISCA. Training manual will be given to the dentists and they will be strongly advised to adhere to the manual for delivering the intervention. Fidelity to intervention delivery will be assessed via audiotaping the interactions between the dentists and patients.
12	How well (actual)	N/A

### Control group

The usual practice, in Pakistan, is no support for tobacco cessation. The participants in the control group will be given self-help material by the dentist, in the form of take-home booklet containing tobacco cessation messages (given in first visit). A TIDierR checklist is provided in Table 2.

**Table 2:** TIDier Checklist-Self-help material.

No	Item	Definition
1	Brief name	Self-help material
2	Why	Self-material has some benefit of increasing quit rates in comparison to no intervention.
3	What (materials)	Take home booklet,(the same booklet given to intervention group).
4	What (procedures)	All patients will be offered the booklet in the 1^st^ visit.
5	Who provided	Dentists
6	How	The booklet will be handed over to the participants by the dentist during the 1^st^ visit.
7	Where	Periodontology and Prosthodontics departments of KCD and SBDC
8	When and How much	1^st^ visit(one booklet)
9	Tailoring	N/A
10	Modification	N/A
11	How well (planned)	N/A
12	How well (actual)	N/A

### Randomization procedure and concealment of allocation

Consented participants will be randomly assigned to the control (self-help material) or intervention arm, in a 1:1 ratio. An independent statistician will prepare a computer-generated randomisation schedule in random-sized permuted blocks. The allocation schedule will be contained in a sealed opaque envelope each bearing on the outside the name of the study site and a unique number for each participant. The sealed envelopes will only be accessible to the research team, only opening them after informed consent and baseline measures have been obtained.

### Blinding

Due to the nature of the intervention, the research team, participants and participating dentists will not be blinded to the assigned intervention.

### Pilot RCT visits and follow-up

Participants will be asked to attend three study visits, each approximately two weeks apart. These will be scheduled by the dentists treating the patient in line with the patient’s treatment. Additionally some of the participants will be asked to make an additional visit for interview ‘phase 3’.

### Withdrawal of study participants

Participants will be free to withdraw from the study at any point without giving a reason and without their legal rights or dental care being affected. However, the information collected will be retained and will be used confidentially for purposes relating to this research study only.

### Data collection and outcome measures

*Phase 1:* Demographic data will be collected from all participants in this phase. Qualitative data will be collected through semi structured, in-depth interviews, which will be audio recorded. The interview topic guide that will be used for interviews with the patients who are ST users, is guided by the Capability- Opportunity-Motivation-Behavior (COM-B) model [25]. The COM-B model recognizes behaviour as a product of an interrelated system which involves an individual’s or group’s capability (physical and psychological), opportunity (social and physical) and motivation (reflective and automatic) [25]. The guide will explore the patients’ ST behaviour (initiation, continued use, cessation attempts) and factors influencing those behaviours. Questions are organized according to the three domains of COM-B model i.e., knowledge of the associated health risks from ST use and skills in managing triggers (capability), access to cessation support via healthcare and the role of other people (opportunity), views and emotions about ST use and cessation (motivation) [17]. Additionally, their views about the contents of BISCA and trial will be assessed as well.

Likewise, dentists working in teaching hospitals have a wide and complex range of factors influencing their behaviour of offering cessation support to their patients in routine clinical practice. The topic guide that will be used for the interviews with the dentists will be guided by the COM-B model. The topic guide will thus enquire into dentist’s capability, the opportunities available to them and the factors influencing their motivation to offering behavioural cessation support in routine [15]. In addition to this, the guide will also include questions, to explore the current practices of the dentists and their views on the intervention (BISCA) and the trial processes. The first author will collect demographic data, conduct the interviews and audio record them.

*Phase 2 & 3*: Demographic information will be collected from all trial participants at baseline. ST use history will be collected using ST use questionnaire, which will be administered at baseline and in all subsequent visits. The Fagerstörm Tobacco and Nicotine Dependency scale for Smokeless Tobacco (FTND-ST) [26], Fagerstörm Test for Nicotine Dependence FTND and The Oklahoma Scale for Smokeless Tobacco Dependence (OSSTD) [27], will be used to measure nicotine dependence amongst participants. These scales will be administered at baseline and 3^rd^ visit.

The visits of the first 10% of the patients (control and intervention) will be audio recorded to, assess fidelity to the intervention and to ascertain cross-over between the two arms. Audio recordings, have an advantage over direct or in-person observation, in that direct observations can be potentially more intrusive and labour intensive and can influence the delivery/implementation [28]. Likewise, self-reports might be less labour intensive compared to audio recordings, however, there are concerns regarding the respondent misunderstanding the question or answering the questions quickly/carelessly [16, 28]. Fidelity, will be ascertained using the fidelity index for BISCA, which consists of 26-items. Of these, 22 items (representing the intervention components) are included in the ‘adherence index’ whereas, six items ‘assessing the competence with which the intervention is delivered’ are included in the ‘quality index’ [16]. The adherence of the participants to the intervention, will be assessed by estimating the completion rates of the calendars, that will be given to the patients at the quit-session.

All patients will be contacted telephonically, by the first author at three and six months after completion of their 3rd visit to assess their self-reported ST use.

Some of the trial participants ‘participating patients and dentists’ will be invited for interview, after intervention delivery, to get an insight into the views and opinions, regarding feasibility and acceptability of the intervention. Information about this will be given in the participant information sheet before recruitment of the trial. Whereas, separate informed consent will be sought by the first author from these participants prior to the interviews.

All personal data will be stored securely in order to protect confidentiality before, during and after the study. Minimal personal data will be collected and recorded on a demographic sheet against the participant IDs. All correspondence details will be deleted once no more needed. All the data will be de-identified using a participant ID. An encrypted audio recorder will be used for audio recording of the interactions between the dentists and the dental patients (participants) as well as interviews with the participants, before and after the trial.

### Outcomes

Outcome measures will include: rates of eligibility, recruitment and retention, the provision of data on; ST use behaviour, self-reported ST use, FTND-ST, OSSTD and self-reported quit rates, fidelity to the intervention and qualitative assessment of acceptability and feasibility.

### Adverse events

No adverse events will be reported for this study.

### Ethics

The study will be conducted in accordance with the principles of the International Conference on Harmonisation Tripartite Guideline for Good Clinical Practice ‘ICH GCP’ and the ‘Declaration of Helsinki’ protocols will be followed. The study has received a favourable ethics opinion from Edinburgh Medical School Research Ethics Committee ‘EMREC’ UoE, Advanced Study Research Board ‘ASRB’ and Ethics Committee of Khyber Medical University and Khyber College of Dentistry, Pakistan.

### Analysis

As recommended, the analysis of this feasibility study will be limited to descriptive statistics to make comparisons between the intervention and control group [23, 29–31]. Baseline data such as dependency scores will be measured as mean and standard deviations (SDs), while categorical data, will be reported as counts and percentages. Similarly, feasibility outcome measures, will be measured as rates/proportions and will be reported with 95% confidence intervals (CIs). Whereas, fidelity index, will be expressed as mean and SD. Quit rates and dependency scores between the two groups will be compared and association between quit status and socioeconomic variables will be determined. All interviews will be, audio-recorded and transcribed verbatim. Thematic analysis will be used to analyse the transcripts, using framework analysis approach. The analysis will be done in Microsoft Excel and NVivo-11 software.

### Dissemination

The findings of this study will be written up in the first author’s university doctoral thesis and presentation, for the thesis defence. A brief summary report of the findings will also be prepared, which will be shared with the participating hospitals and with the participants upon request. Findings will also be submitted for publication to an international, peer-reviewed journal.

## Discussion

Tobacco use is the single most preventable cause of premature death and disability. Smoking shortens life by an average of 10 years [32], whereas, the use of ST alone accounts for 9% of all deaths and 23% of disability-adjusted life years ‘DALYS’ attributable to tobacco use [4]. The South-East-Asian region bears the greatest toll of ST-related mortalities and ST-related ‘DALYS’ [4, 33]. Indeed, it is difficult to identify any other risk factor that singularly presents such ‘a mix of lethality, prevalence, and neglect’, despite availability of evidence-based, cost-effective interventions [34].

ST users are exposed to more than 28 different carcinogens. The level and type of nicotine that the product contains and the level of carcinogens, such as, tobacco-specific nitrosamines, nitrite, nitrate and heavy metals such as nickel, cadmium and chromium, defines the product’s toxicity. Amongst the diseases associated with ST use, the greatest evidence is for oral cancers. ST use has been reported as the single most important risk factor in the all-cause mortality of oral cancer [4]. Despite the substantial overall progress in the implementation of FCTC measures, ST control considerably lags behind. Widely promoted as a less harmful substitute for cigarette smoking and essentially considered as a problem exclusive to the South-Asian region, ST has received very limited attention from policymakers, even in regions with a high prevalence of use [35]. The prevalence of ST use in Pakistan is one of the highest in the region. The use of naswar, is associated with an 11 times increased risk of oral cancer [36]. Despite convincing evidence on the harms associated with ST use, the users are offered negligible cessation support.

There is good quality evidence from systematic reviews, to suggest, that behavioural support is effective in increasing quit rates. Behavioural support for tobacco cessation includes; advice, counselling, motivation and identification of strategies to cope with withdrawal symptoms [37]. There exists a great variation in the content and delivery of behavioural interventions for tobacco cessation [38]. Typically, the interventions involve; offering advice to quit tobacco use, providing information on how to quit, or a combination of both. However, the strategies that are used to achieve these objectives differ. These interventions may be offered to tobacco users who are motivated to quit or to all users, irrespective of their intention to quit. Pharmacotherapy, may or may not be provided in combination with behavioural interventions. The intervention could be offered as a one-time brief advice or as more intensive, multiple sessions.

In line with the guidelines of the WHO FCTC article 14, it is recommended that all oral healthcare professionals, provide support for tobacco cessation, to all patients, by delivering at least brief tobacco cessation interventions. While there is good quality evidence to suggest that behavioural interventions, delivered by oral healthcare providers can effectively increase quit rates amongst tobacco users [38, 39], most of this evidence comes from high-income countries which clearly limits the generalizability of the findings and warrants the need for more evidence on the feasibility and effectiveness of these interventions especially in LMICs. The findings of this study will help to explore whether a structured behavioural support intervention, is feasible and acceptable for dental patients who are ST users and how feasible it is for dentists to deliver this within routine clinical work in a low-middle income country. Additionally, this study will provide the essential pre-requisite required for conducting a definitive trial on the provision of behavioural support via dentists in clinical settings in LMICs. While this will be the first study using a randomized controlled design to assess the feasibility and acceptability of a structured behaviour support programme for ST cessation delivered via dentists in routine dental practice in Pakistan, there are some foreseeable limitations. Owing to the nature of the intervention, it is not possible to blind the patients, dentists and the researcher. All qualitative interviews, will be conducted by the first author, who is a female dentist. A potential limitation is that male patients might not be comfortable talking about their habit with a female interviewer. Conversely, female respondents may react favourably to being interviewed by a female. The interviewer is also a dentist and this could influence the respondents comfort at talking freely. In order to reduce this influence, the interviewer will not be involved in the provision of their care and they will be reassured that anything they say in the interview will not affect their treatment in any way. Similarly, for the third phase, the patients, as well as the dentists, might not openly share their views on the feasibility and acceptability of the intervention. Given that the study is led by the first author, the respondents might not voice their concerns with the interviewer as they would with someone who is not part of the recruitment and intervention delivery team. Key strengths and limitations of this study have been highlighted in Box 1.

Box 1 Strengths and limitations of this study

- First study using a randomised controlled design to assess the feasibility and acceptability of a structured behaviour support programme for smokeless tobacco cessation delivered via dentists in routine dental practice in Pakistan.
- Evidence of implementing behaviour cessation via dentists in Pakistan will be established.
- This study will address an important evidence gap regarding the feasibility of dentists in tobacco cessation in a low middle income country.
- The multicentre design including patients from two tertiary level teaching hospitals in Pakistan comparing two feasible strategies will support external validity and implementation.
- Owing to the type of interventions, blinding of the patients and intervention providers is not possible.
